# Ultrasound Use in Clinical Decision-Making for Spasticity and Contracture Management in Neurological Rehabilitation

**DOI:** 10.3390/jfmk11040405

**Published:** 2026-10-03

**Authors:** Nicola Theis, Cherry Kilbride, Stephen A. Ashford

**Affiliations:** 1 School of Education, Health and Science, University of Gloucestershire, Gloucester GL2 9HW, UK; ntheis@glos.ac.uk; 2 Department of Health Sciences, Brunel University of London, Middlesex UB8 3PN, UK; 3 Royal Free London NHS Trust, London NW3 2QG, UK; 4 Department of Palliative Care, Policy and Rehabilitation, Cicely Saunders Institute, Florence Nightingale Faculty of Nursing, Midwifery and Palliative Care, King’s College London, London SE5 9PJ, UK; stephen.ashford@nhs.net; 5 Regional Hyper-Acute Rehabilitation Unit, Northwick Park Hospital, London North West University Hospitals, London HA1 3UJ, UK

**Keywords:** spasticity, contracture, ultrasound

## Abstract

Ultrasound is increasingly used in neurological rehabilitation, most visibly to improve the accuracy and safety of botulinum toxin (BoNT) and phenol administration for spasticity. However, its potential to contribute to wider clinical decision-making remains underdeveloped, particularly through characterisation of structural muscle–tendon unit (MTU) changes that may help clinicians interpret the relative neural and structural contributors to stiffness and contracture. This position paper argues that ultrasound should be conceptualised not only as a procedural adjunct but also as a clinical decision-support tool. We synthesise current evidence supporting muscle ultrasound for tissue characterisation and intervention guidance, outline barriers to implementation, and distinguish established procedural applications from the emerging use of ultrasound for tissue-informed clinical decision support. The modified Heckmatt scale is presented as one feasible method for tissue characterisation within this broader ultrasound toolkit, while recognising that validated thresholds linking ultrasound findings to specific treatment decisions have not yet been established. Further methodological standardisation, competency development and prospective evaluation are required before these emerging applications can be incorporated consistently into routine clinical pathways.

## 1. Introduction

Stroke and other forms of acquired brain injury (ABI) can result in neurological and musculoskeletal impairments that affect motor control and muscle structure [1,2]. Following injury to the central nervous system, disruption of descending motor pathways reduces voluntary motor activation and contributes to muscle disuse, triggering structural and functional changes within the muscle–tendon unit (MTU) [2,3]. Muscle atrophy develops as a consequence of reduced muscle activation and inactivity, while progressive alterations in the extracellular matrix contribute to fibrosis and reduced tissue elasticity [4]. Fatty infiltration may replace contractile tissue and further impair muscle quality [5]. Post-stroke adaptations in muscle architecture and mechanical properties, including alterations in fascicle length and muscle stiffness, have also been documented [6,7]. Collectively, these neurological and musculoskeletal changes contribute to impairments in motor function, mobility and activity following stroke [8,9].

These structural adaptations contribute to the development of contracture [10,11]. Contracture refers to a loss of passive joint range of movement associated with structural shortening or remodelling of the muscle–tendon unit and surrounding periarticular tissues [11]. Throughout this paper, several terms describing increased resistance to movement are used and should be distinguished. Spasticity is defined as disordered sensorimotor control resulting from an upper motor neuron lesion, presenting as intermittent or sustained involuntary activation of muscle [12]. Rigidity refers to an increased resistance to passive movement that is classically considered relatively independent of movement velocity and is associated predominantly with extrapyramidal disorders such as Parkinson’s disease [13]. Stiffness is a biomechanical property describing the resistance of a tissue or structure to deformation and is commonly expressed as the change in force relative to the resulting change in length or displacement [14]. Although these phenomena may coexist, they represent distinct impairments and should not be used interchangeably.

Contractures are common following moderate to severe stroke and are associated with poorer functional outcomes. In a prospective cohort of adults admitted to hospital following stroke, approximately half developed at least one contracture within six months [15]. Other longitudinal studies have demonstrated that contracture may develop early after stroke and become increasingly apparent over subsequent weeks and months [16,17]. The mechanisms and sequence of events leading to contracture development remain incompletely understood [10,11,18]. Longitudinal findings from the Care of the Arm after STroke (CAST) study demonstrate that impairments including hypertonicity and loss of range of movement remain clinically relevant during the first year following severe stroke [19]. Contracture development is complex and multifactorial, involving changes to muscles, tendons, ligaments and joint capsules [11], and is influenced by factors including the severity of motor impairment, mobility and functional recovery [15,17,18]. Importantly, contracture is a structural musculoskeletal complication that may occur across a range of neurological conditions, irrespective of whether increased muscle tone is characterised by spasticity or rigidity. Although spasticity and contracture frequently coexist following stroke, they are distinct clinical entities and should not be considered synonymous [18,20]. Distinguishing neural and structural contributors to impaired movement is therefore important for appropriately targeted management [11,21].

The clinical and economic burden associated with contractures is considerable. Among individuals with acquired brain injury receiving specialist inpatient rehabilitation, those with contractures remained in hospital for an average of 31 days longer than those without contractures, with an additional mean episode cost of £25,588 [22]. Despite this burden, evidence supporting effective prevention and management of contractures remains limited [23,24].

Against this background, ultrasound is becoming increasingly accessible within neurological rehabilitation and is established for selected applications, particularly image-guided interventions for spasticity management [25]. Its use for tissue characterisation and clinical decision support is less established. An important clinical challenge in stroke and ABI is that increased resistance to passive movement may be attributed predominantly to spasticity despite reflecting a combination of neural hyperexcitability and non-neural musculoskeletal changes, including muscle shortening, altered tissue composition and established contracture [11,20,21]. Failure to recognise the relative contribution of these mechanisms may result in interventions being poorly aligned with the underlying impairment. For example, where structural muscle–tendon changes contribute substantially to passive stiffness, interventions addressing muscle length, tissue extensibility or orthopaedic management may need to be considered alongside those targeting neural overactivity. Improving understanding of the relative neural and structural contributions is therefore important for appropriately targeted clinical decision-making.

This position paper considers the role of ultrasound across the continuum from established procedural guidance to the emerging use of tissue characterisation in clinical decision support for spasticity and contracture management. It examines how ultrasound-derived information about muscle structure and composition may complement clinical assessment, while recognising that these applications are at different stages of evidence development and implementation. The modified Heckmatt (mHeckmatt) scale (Table 1) is presented as one potential approach to tissue characterisation within this broader ultrasound toolkit, alongside qualitative visual assessment, quantitative echogenicity analysis and, where available, elastography [26,27]. The paper subsequently considers the evidence supporting these applications, their implications for clinical reasoning, and the training, governance and research requirements necessary for their safe and reproducible implementation.

## 2. Ultrasound in Spasticity and Contracture Management: From Procedural Guidance to Clinical Reasoning

Muscle ultrasound is an established method for assessing muscle structure and can provide information regarding muscle thickness, architecture and tissue composition at the point of care [26,28]. Broadly, ultrasound may contribute to neurological rehabilitation in three ways: (1) identifying muscles and adjacent neurovascular structures during image-guided interventions; (2) characterising structural muscle changes, including changes in echogenicity, muscle composition and architecture; and (3) monitoring tissue response to interventions over time. These applications span, but should not be conflated with, the formal scopes of ultrasound practice discussed in Section 4. While ultrasound guidance for interventions is well established within spasticity services, its role in tissue characterisation and clinical decision support is less developed and less consistently integrated into routine practice.

**Figure 1:**
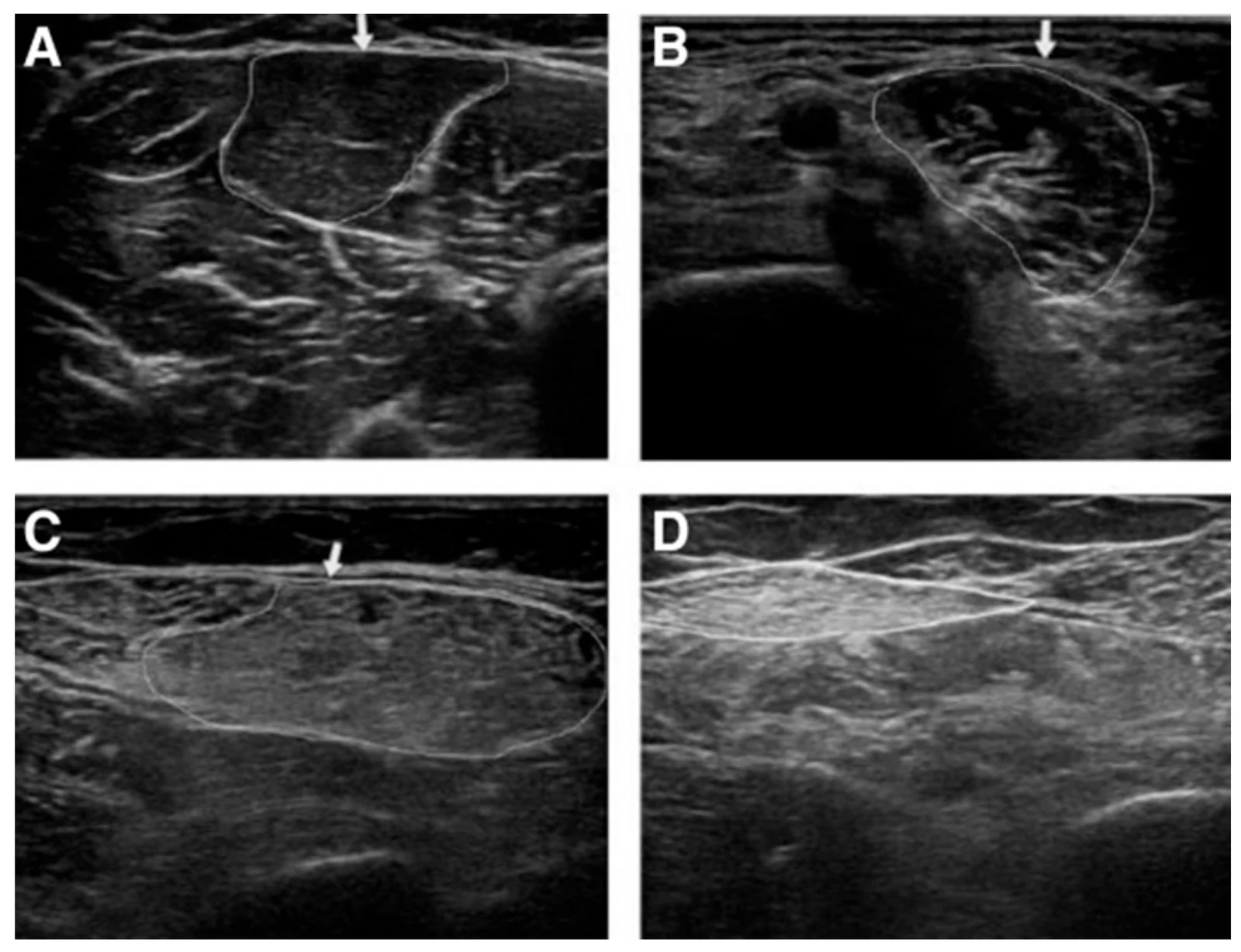
Traced muscles (white arrow) demonstrating the Modified Heckmatt grades. (**A**) Grade 1: normal echogenicity in more than 90% of the muscle that is distinct from bone echo. (**B**) Grade 2: increased muscle echogenicity in 10% to 50% of tissue, but with distinct bone echo and some areas of normal muscle echo. (**C**) Grade 3: marked increase in muscle echogenicity between 50% and 90% of tissue with reduced distinction of bone echo from muscle. (**D**) Grade 4: very strong muscle echogenicity, with near complete loss of distinct bone echo from muscle in more than 90% of tissue. Moreta, Fleet, Reebye, McKernan, Berger, Farag, Munin. Reliability and Validity of the Modified Heckmatt Scale in Evaluating Muscle Changes With Ultrasound in Spasticity, Archives of Rehabilitation Research and Clinical Translation 2020, 2, 4 [30]. Reproduced under a Creative Commons license.

Assessment of muscle echogenicity may provide additional information when increased resistance to passive movement reflects structural muscle–tendon changes alongside, or rather than predominantly, neural mechanisms. Echogenicity describes the brightness of muscle tissue on ultrasound images and reflects the degree of sound wave reflection, with increased echogenicity commonly associated with fibrosis, fatty infiltration and muscle degeneration. The modified Heckmatt (mHeckmatt) scale provides a clinically feasible method for grading muscle echogenicity in individuals with spasticity, bridging qualitative visual assessment and more technically demanding quantitative grayscale analysis [29,30]. The scale uses a four-point ordinal grading system ranging from Grade 1 (normal echogenicity) to Grade 4 (severe muscle degeneration with marked hyperechogenicity), with the modified version improving discrimination between intermediate grades by considering the proportion of muscle demonstrating increased echogenicity [29,30] (Table 1). The mHeckmatt scale has demonstrated good validity and reliability when compared with quantitative grayscale analysis [30] (Figure 1).

Importantly, ultrasound should be considered an adjunct to, rather than a replacement for, comprehensive clinical assessment. Clinical examination remains essential for evaluating neurological impairment through assessment of passive range of movement, resistance to passive stretch at different velocities, muscle length, functional performance and patient history. Clinical measures such as the Modified Ashworth Scale and Modified Tardieu Scale provide information regarding increased resistance to movement but cannot independently distinguish the relative contributions of neural hyperexcitability and structural muscle–tendon pathology. Ultrasound may complement these assessments by providing additional information about muscle quality, architecture and tissue composition. Considered alongside clinical findings, this information may contribute to a more informed interpretation of the relative neural and structural contributors to impaired movement, with potential implications for prognosis and treatment planning.

Beyond echogenicity assessment, ultrasound offers additional approaches to characterising muscle mechanical properties. Shear wave elastography estimates tissue stiffness by measuring the propagation speed of mechanically induced shear waves through muscle. Studies in post-stroke upper limb muscles have demonstrated that elastography-derived measures can be obtained reliably and are associated with clinical measures of spasticity and functional performance [31]. A systematic review similarly concluded that elastography provides useful quantitative information regarding the mechanical properties of spastic muscle, although further methodological standardisation is required before widespread clinical implementation [32,33]. Campanella et al. demonstrated changes in elastography-derived measures associated with spastic dystonia and response to botulinum toxin treatment [34]. In the same study, mHeckmatt grades were positively associated with elastography-derived muscle stiffness in shortened muscles, suggesting that echogenicity and elastography may provide complementary information regarding MTU characteristics [34]. Moreta et al. similarly demonstrated excellent inter- and intra-rater reliability of the mHeckmatt scale and a strong relationship between qualitative grading and quantitative grayscale analysis [30]. Together, these findings support the potential utility of complementary qualitative and quantitative ultrasound approaches for tissue characterisation, while highlighting the need for further methodological and clinical validation [30,32,35].

However, evidence supporting the measurement properties of the mHeckmatt scale should be distinguished from evidence supporting its use as a clinical decision tool. At present, validated thresholds linking individual mHeckmatt grades to specific interventions or predicting treatment response have not been established. The scale should therefore be used as an adjunct to comprehensive clinical assessment rather than as a stand-alone determinant of treatment selection. Establishing clinically meaningful thresholds and prospectively evaluating whether and how ultrasound-derived measures contribute to treatment decision-making represents an important priority for future research.

Collectively, these approaches support an emerging role for ultrasound that extends beyond procedural guidance towards tissue characterisation and clinical decision support. Ultrasound-derived information about muscle quality and MTU characteristics may complement clinical examination and contribute to clinical reasoning, goal setting, intervention planning and monitoring. However, evidence demonstrating that incorporation of these measures into clinical pathways improves treatment selection or patient outcomes remains limited. Further validation is therefore required to establish how ultrasound-derived information should most appropriately contribute to clinical decisions.

## 3. Ultrasound-Guided Interventions: Established Procedural Applications

Ultrasound guidance for botulinum toxin (BoNT) injection has the strongest evidence base and is the most widely adopted application of ultrasound within spasticity management. The rationale is straightforward: if the intended muscle is not accurately targeted, treatment efficacy may be reduced and unintended weakness of adjacent muscles may compromise function. Multiple studies have demonstrated that ultrasound guidance improves localisation accuracy compared with anatomical landmark-based approaches, particularly when targeting smaller or deeper muscles and anatomically complex regions. Ko et al. reported markedly higher injection accuracy with ultrasound guidance than with non-guided techniques, with particularly large differences observed in more challenging muscle groups [36]. Earlier work on forearm muscles similarly demonstrated improved localisation while reducing the risk of inadvertent injection into adjacent vascular or neural structures [37]. More recent evidence continues to support improved procedural accuracy with ultrasound guidance, even following relatively limited training [38].

Beyond BoNT, ultrasound also supports other interventional procedures used in neurological rehabilitation. Phenol neurolysis remains an important option for focal spasticity management, particularly when longer-lasting denervation is required or when response to BoNT is limited. Matsumoto et al. demonstrated that combining ultrasound with electrical stimulation improved targeting during musculocutaneous nerve phenol neurolysis for elbow flexor spasticity [39], while ultrasound-guided phenol neurolysis has also been reported to be both feasible and safe in people with spinal cord injury [40]. Similarly, ultrasound, commonly combined with electrical nerve stimulation, has been used to guide cryoneurolysis by facilitating localisation of peripheral nerves and motor branches. Emerging observational studies and case series have reported favourable outcomes including reductions in spasticity, improvements in range of movement and goal attainment, although controlled evidence remains limited [41,42,43]. Collectively, these studies support ultrasound as an important procedural tool for interventions where precision and safety are essential.

Although ultrasound guidance consistently improves procedural accuracy, improvements in targeting precision do not necessarily translate into proportional gains in clinical or functional outcomes. Santamato et al. reported improved clinical outcomes following ultrasound-guided BoNT injections compared with anatomically guided injections [44], and a clinical discussion paper argued that accumulating evidence supports routine ultrasound guidance within spasticity services, particularly when targeting muscles where anatomical landmark accuracy is limited [45]. However, treatment response is influenced by numerous factors beyond injection accuracy, including patient selection, chronicity of impairment, the severity of underlying muscle–tendon structural change, rehabilitation intensity and functional goals [46,47]. Ultrasound guidance should therefore be regarded as an important component of optimising intervention delivery rather than a guarantee of superior clinical effectiveness.

Importantly, procedural accuracy addresses only one component of clinical decision-making. Ultrasound-guided injection can confirm identification of the intended target and support safe needle placement but does not, in isolation, establish the relative contribution of neural hyperexcitability and structural muscle–tendon changes to increased resistance to movement. As discussed in Section 2, ultrasound assessment of muscle architecture, echogenicity, thickness and other tissue characteristics may provide complementary information about MTU status, although the clinical interpretation and application of these measures remain less well established than procedural ultrasound guidance.

This distinction between established procedural guidance and the emerging use of ultrasound for tissue characterisation has important implications for scope of practice, training and governance. The following section therefore considers ultrasound practice according to defined clinical scopes, distinguishing the competencies and governance requirements associated with procedural applications from those required for tissue characterisation and clinical decision support.

## 4. Scope of Practice, Competency, and Governance: Aligning Ultrasound Use to Clinical Risk and Purpose

As ultrasound becomes increasingly portable and more widely used by clinicians outside radiology, clearly defined scopes of practice are essential to support patient safety, appropriate interpretation and consistent clinical standards. The term *ultrasound use* encompasses a spectrum of activities associated with different levels of complexity, training requirements and clinical risk. The point-of-care ultrasound (PoCUS) literature consistently emphasises the importance of formal education, competency assessment and appropriate governance structures, as confidence without competence may lead to image misinterpretation and inappropriate clinical decision-making [25,48].

An international Delphi study linked to the development of the UK National Framework for Ultrasound Practice proposed a structured framework describing three progressive scopes of ultrasound practice within spasticity management [25] (Table 2). The first two scopes relate primarily to ultrasound-guided interventions, while the third extends ultrasound into evaluation of muscle structure and composition to support clinical assessment and decision-making. Although originally developed in the context of spasticity interventions, the underlying principles of competency, governance and clinical responsibility have wider relevance to neurological rehabilitation.

Importantly, these scopes are at different stages of evidence development and clinical implementation. Ultrasound-guided procedural practice has a comparatively established evidence base, whereas the use of ultrasound-derived tissue characteristics to directly inform treatment selection remains an emerging area. Evidence supporting individual measures such as echogenicity, muscle architecture and elastography varies in study design, population and outcome, and validated thresholds linking these measures to specific clinical decisions have not yet been established. The evidence and governance requirements for each scope should therefore be considered in relation to its clinical purpose and maturity.

For the procedural scopes (Scopes 1 and 2), the principles established within the original framework have subsequently been translated into practice recommendations and an applied governance framework. Ashford et al. [48] describe implementation of ultrasound image-guided BoNT injection in clinical practice, including training, competency development, governance, documentation and quality assurance. Since publication of the original framework, ultrasound-guided cryoneurolysis has also emerged as an intervention consistent with the procedural principles represented within Scope 2. Readers are directed to Ashford et al. [48] and the existing framework [25] for detailed operational guidance relating to procedural ultrasound practice; the present paper does not seek to duplicate these recommendations.

Scope 3 represents a less developed extension of ultrasound practice. It moves beyond procedural guidance towards tissue characterisation and the potential use of ultrasound-derived information to support clinical reasoning. Assessment of muscle echogenicity, architecture and, where available, elastography may provide additional insight into the structural status of the MTU. However, direct evidence which demonstrates that incorporating these measures into clinical pathways improves treatment selection or patient outcomes, remains limited. Furthermore, operational standards for image acquisition and interpretation, competency requirements and clinically meaningful thresholds are less well established than for procedural ultrasound. Table 3 therefore summarises the current evidence supporting the principal applications within Scope 3, together with their limitations and priorities for further development.

**Figure 2:**
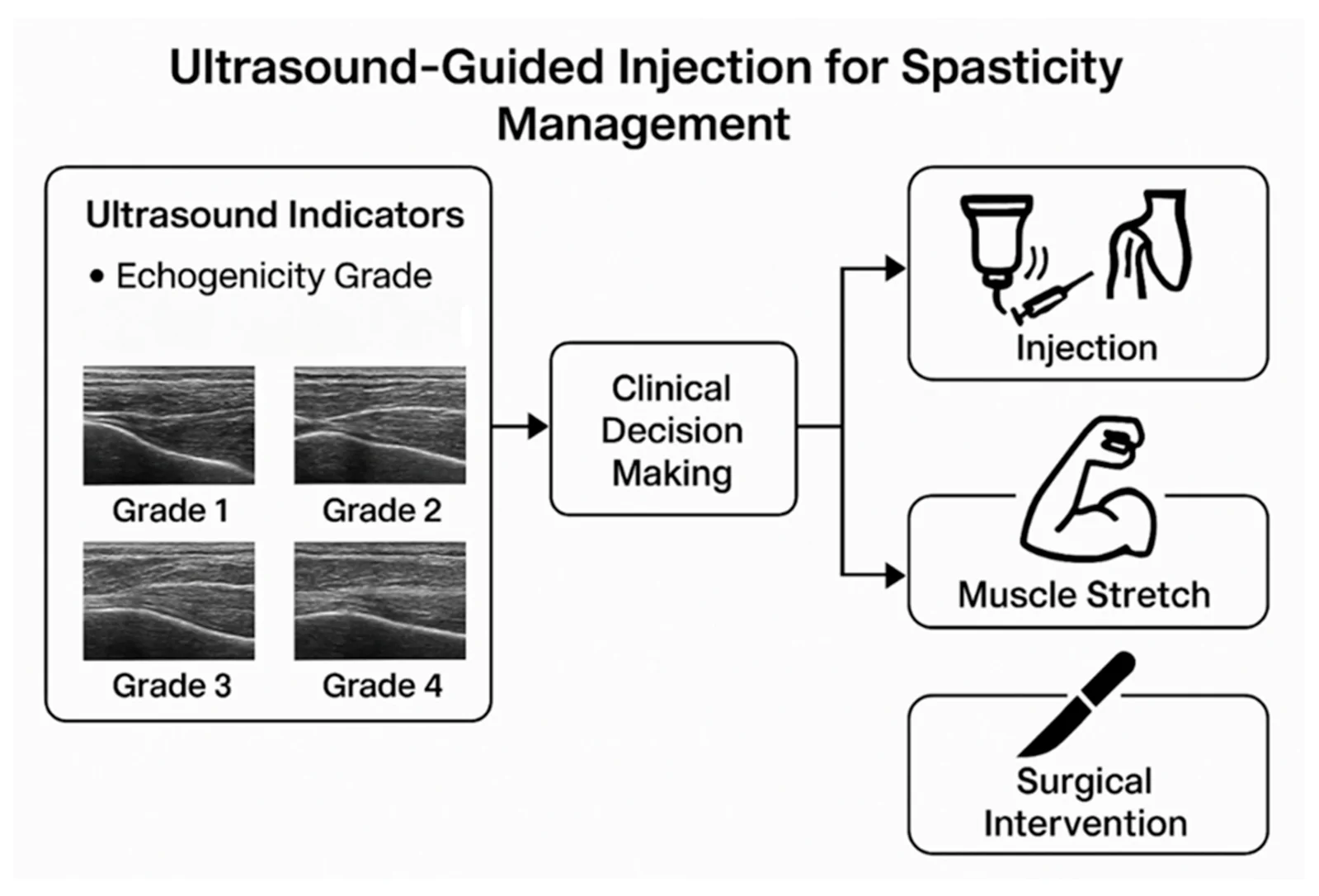
Conceptual integration of muscle ultrasound findings within multidisciplinary clinical decision-making. *This figure represents a conceptual framework rather than a validated clinical decision algorithm. No validated mHeckmatt thresholds currently exist for directing specific treatment choices.* Image created using Microsoft Copilot 2026.

The potential contribution of Scope 3 can be illustrated through clinical reasoning. Two patients may present with similar resistance to passive elbow extension on clinical examination, while ultrasound demonstrates different structural characteristics of the target muscle. One may show relatively preserved architecture and low echogenicity, whereas another may demonstrate marked hyperechogenicity, reduced muscle thickness and shortened fascicles. These findings should not be interpreted as stand-alone indicators for particular interventions; rather, they provide additional information that can be considered alongside passive range of movement, resistance to passive stretch, pain, function, patient goals and other clinical findings. In this way, ultrasound may contribute to consideration of the relative neural and structural components of impairment and to multidisciplinary treatment planning (Figure 2). Neural and structural impairments frequently coexist, and ultrasound should therefore complement, rather than replace, comprehensive clinical assessment.

Evidence supporting this broader role is emerging. Moreta et al. demonstrated that the modified Heckmatt scale provides a reliable method of evaluating pathological muscle changes and correlates with quantitative grayscale analysis [30]. Campanella et al. and Heckmatt et al. demonstrated relationships between muscle echogenicity and ultrasound-derived measures of tissue characteristics, supporting the potential complementary use of qualitative and quantitative approaches [29,34]. However, as outlined in Section 2 and Table 3, evidence demonstrating that these measures can be translated into validated treatment thresholds or decision rules remains limited. Ashford et al. [25] proposed structured spasticity pathways in which ultrasound findings are integrated with clinical assessment and treatment planning rather than interpreted as an isolated assessment. Figure 2 builds on this principle by illustrating the conceptual integration of muscle ultrasound within multidisciplinary clinical reasoning; though is not a validated clinical decision algorithm yet.

The differing maturity and clinical purpose of the three scopes also have implications for governance. For procedural ultrasound, established frameworks provide a basis for training, competency assessment and quality assurance [25]. For Scope 3, governance must evolve alongside the evidence base and should encompass not only competency in image acquisition but also appropriate interpretation, documentation, reporting and integration of ultrasound findings with comprehensive clinical assessment. Development of reproducible acquisition methods, competency standards and validated approaches to clinical interpretation therefore represents an important priority before Scope 3 can be implemented consistently across services.

## 5. Barriers to Implementation: Why Adoption Remains Uneven Despite Availability

Despite increasing availability of ultrasound devices, implementation barriers remain across spasticity and neurological rehabilitation services. In a Canadian survey of rehabilitation physicians, limited familiarity with point-of-care ultrasound (PoCUS) and uncertainty regarding its clinical value were identified as key obstacles to integrating ultrasound into routine spasticity management [59]. These findings mirror broader evidence surrounding implementation within stroke rehabilitation, where organisational and clinician-level barriers—including limited time and resources, knowledge and skills gaps, and scepticism regarding new approaches—continue to impede the adoption of evidence-based practice [60]. Ultrasound implementation is particularly vulnerable to these challenges because successful integration requires not only access to equipment but also appropriate technical standards, sustained education, supervised clinical experience and robust governance.

An additional challenge, particularly for Scope 3 practice, is the lack of sufficiently standardised acquisition, analysis and reporting protocols across muscle ultrasound applications. Measures of echogenicity, muscle architecture and elastography may be influenced by factors including probe orientation and pressure, anatomical site, machine settings, device characteristics and image-processing procedures. Although methodological recommendations exist for individual techniques, consensus minimum standards specifically for their application within neurological clinical decision-making have not yet been established. This variability has implications for reproducibility, interpretation and comparison of findings between clinicians and services. Development and validation of pragmatic standards for image acquisition, analysis and reporting are therefore important prerequisites for consistent implementation of tissue characterisation within routine neurological rehabilitation.

Workforce and training requirements present a further barrier. Evidence from the wider PoCUS literature indicates that clinicians commonly report insufficient education, limited opportunities for supervised practice and a lack of clearly defined competency and credentialling pathways, even where ultrasound technology is readily available [61]. Although medical education programmes demonstrate that ultrasound skills can be successfully taught, national surveys continue to report considerable variability in curriculum provision, inconsistent competency standards and limited faculty expertise [62]. These challenges are particularly relevant when ultrasound practice extends beyond procedural guidance into tissue characterisation and clinical decision support, where clinicians must develop competency not only in image acquisition but also in interpretation and integration of findings with the wider clinical assessment.

A further challenge is demonstrating clinical value. The benefits of ultrasound-guided interventions are relatively easy to appreciate because improvements in procedural safety and targeting accuracy are immediately observable. By contrast, the value of ultrasound-based tissue characterisation is less tangible because its principal contribution lies in supporting clinical reasoning rather than directly delivering treatment. Ultrasound has the potential to improve differentiation between neural and structural contributors to movement impairment and thereby support more appropriate treatment selection; however, robust evidence demonstrating improved patient outcomes, cost-effectiveness or healthcare savings remains limited. Even where tools such as the modified Heckmatt scale demonstrate good measurement properties, clinicians may remain uncertain about how ultrasound findings should influence treatment selection, intervention timing or escalation of care [30]. This may create a cycle in which limited clinical adoption generates relatively little real-world evidence of impact, which in turn reinforces uncertainty regarding its value and further limits implementation.

## 6. Overcoming Barriers: Strengthening Pathways, Training and Quality Systems

Successful implementation of ultrasound within neurological rehabilitation requires a coordinated strategy combining pathway integration, education, competency development and governance. Structured protocols and standardised documentation have been associated with improved uptake of point-of-care ultrasound (PoCUS) in clinical practice, with studies of ultrasound competency programmes demonstrating sustained improvements in use following the introduction of structured training and quality assurance systems [63]. Within spasticity and neurological rehabilitation services, ultrasound should therefore be incorporated into existing assessment and treatment pathways rather than introduced as a standalone investigation. Routine documentation could integrate conventional clinical measures, including passive range of movement, resistance to passive movement, pain and functional goals, with relevant ultrasound findings such as modified Heckmatt grade, muscle thickness, echogenicity and other indicators of tissue quality. Importantly, documentation should record not only the ultrasound findings themselves but also how these findings contribute to clinical interpretation and subsequent treatment planning. This would support a shift from ultrasound as an isolated imaging procedure towards its use as a component of structured clinical decision-making.

Education and training should be aligned with the defined scope of practice. Expanding PoCUS within neurological rehabilitation requires clinicians to develop competencies appropriate to their intended application, ranging from ultrasound-guided interventions to muscle assessment, image interpretation and integration of findings into clinical reasoning. Evidence indicates that structured ultrasound education can improve competency and promote sustained clinical use. Clinicians undertaking formal ultrasound training have continued to incorporate PoCUS into practice several years after training, highlighting the importance of supervised learning and ongoing clinical exposure in maintaining competence [64]. Similarly, longitudinal integration of ultrasound training within medical curricula has been shown to support skill retention and routine clinical use more effectively than isolated short courses [65]. Within spasticity management, Ashford et al. have also highlighted the value of structured training packages in improving clinician confidence and consistency in assessment and treatment planning [25]. A staged approach to ultrasound competency development, incorporating theoretical education, supervised clinical practice, formal competency assessment and continuing professional development, is therefore likely to be necessary to support safe and effective implementation.

Implementation must also be supported by an appropriate workplace culture and quality assurance infrastructure. Evidence from implementation research highlights the importance of local clinical champions, integration into established workflows and continuous quality improvement in sustaining new clinical practices [66]. Within ultrasound services, regular audit, peer review and quality assurance processes can support consistency, accuracy and continuing professional development [67]. For neurological rehabilitation services, this could include periodic review of ultrasound-guided intervention practice, peer review of tissue characterisation findings and audit of how ultrasound findings are incorporated into documented clinical reasoning and treatment pathways. Linking ultrasound findings with subsequent interventions and patient outcomes would additionally provide services with opportunities to evaluate whether implementation is meaningfully influencing clinical practice.

Emerging technologies may further support implementation. Artificial intelligence (AI)-assisted ultrasound tools have the potential to support image acquisition and interpretation and reduce operator variability [68]. Although these technologies cannot replace clinical reasoning, professional judgement, appropriate training or governance, they may help facilitate wider adoption by supporting more consistent acquisition and interpretation, particularly where access to specialist ultrasound expertise is limited. Their role within neurological rehabilitation will nevertheless require appropriate validation and evaluation before routine implementation.

Future evaluation of Scope 3 should extend beyond technical validity and reliability to determine whether ultrasound-informed assessment changes clinical decision-making and, ultimately, improves patient outcomes. Relevant outcomes may include agreement and reproducibility of ultrasound findings; changes in treatment selection, timing or escalation; goal attainment; passive and active function; pain; range of movement; and the feasibility and acceptability of ultrasound-informed pathways. Service-level outcomes, including assessment time, pathway utilisation and resource requirements, should also be considered. Economic evaluation will be necessary before claims regarding cost-effectiveness or healthcare savings can be substantiated. These outcomes should be evaluated prospectively as Scope 3 pathways, standardised protocols and competency frameworks are developed and implemented.

Ultimately, successful implementation will depend not simply on wider availability of ultrasound technology, but on establishing the clinical infrastructure required to use it meaningfully. Standardised acquisition and reporting, scope-specific competency, appropriate governance and explicit integration of ultrasound findings into clinical reasoning will all be necessary if ultrasound is to progress beyond procedural guidance towards a broader role in neurological rehabilitation. Ultrasound should therefore be viewed as an adjunct to comprehensive neurological and musculoskeletal assessment that can provide additional information to support, rather than replace, clinical judgement.

## 7. Summary and Position Statement

To date, the principal clinical application of ultrasound within spasticity management has been procedural, improving the accuracy and safety of interventions including botulinum toxin, phenol neurolysis and cryoneurolysis. However, ultrasound also provides clinically relevant information about the structural and mechanical properties of the muscle–tendon unit, including muscle echogenicity [30], tissue stiffness [31,33] and changes following intervention [34]. These capabilities provide a rationale for extending ultrasound beyond procedural guidance towards a broader role in supporting clinical assessment and decision-making within neurological rehabilitation.

Consistent with Ashford et al. [25], this position paper proposes that ultrasound should be incorporated within structured clinical reasoning frameworks and care pathways, supported by clearly defined scopes of practice, competency standards and governance systems. Within this framework, tools such as the modified Heckmatt scale should be considered one component of a broader ultrasound toolkit rather than standalone diagnostic measures. When interpreted alongside clinical examination, patient history and functional goals, ultrasound findings may provide additional information to help clinicians distinguish the relative contribution of neural and structural factors to movement impairment and inform treatment planning.

The evidence base supporting this role differs across the proposed scopes of practice. Ultrasound-guided interventions have a comparatively established evidence base, whereas the use of ultrasound tissue characterisation to directly inform treatment selection remains an emerging area. Before widespread adoption of Scope 3 practice, further work is required to establish standardised acquisition and reporting protocols, appropriate competency frameworks and, importantly, whether ultrasound-informed assessment meaningfully changes clinical decisions and improves patient outcomes.

Our position is therefore not that ultrasound should replace established clinical assessment, nor that individual ultrasound findings should determine treatment in isolation. Rather, ultrasound should be developed as an adjunct to comprehensive neurological and musculoskeletal assessment, providing additional information about MTU structure and mechanical properties that may strengthen clinical reasoning. With appropriate standardisation, training, governance and prospective evaluation, this broader application has the potential to support more individualised, mechanism-informed management of spasticity and contracture across neurological rehabilitation.

## Figures and Tables

**Table 1:** Comparison of the Heckmatt and mHeckmatt Scales.

Grade	Original Heckmatt Scale	Modified Heckmatt Scale
1	Normal	Normal echogenicity in more than 90% of the muscle that is distinct from bone echogenicity.
2	Increased muscle echogenicity intensity with distinct bone echogenicity.	Increased muscle echogenicity in 10–50% of tissue with reduced distinction of bone echogenicity from muscle.
3	Marked increase in muscle echogenicity with a reduced bone echogenicity.	Marked increased in muscle echogenicity between 50% and 90% of tissue with a reduced distinction of bone echogenicity from muscle.
4	Very strong muscle echogenicity and complete loss of bone echogenicity.	Very strong muscle echogenicity with near complete loss of distinct bone echogenicity from muscle in >90% of tissue.

**Table 2:** Scope of practice statements for ultrasound use within spasticity management.

Scope of Practice Statements
1. Visualization and identification of target muscle and neighbouring tissues (including neurovascular structures). Visualization of needle passage and subsequent localization of botulinum toxin into target muscle; avoiding at risk structures. *The first scope is procedural guidance for BoNT injection: this requires image optimisation, accurate identification of muscle boundaries, real-time needle visualisation, and safety awareness around neurovascular structures.*
2. Visualization and identification of target muscle or nerve; and neighbouring tissues. Visualization of needle passage and subsequent localization of injectate such as phenol (or cryoneurolysis) * into target tissue; avoiding at risk structures. *The second scope is procedural guidance for phenol neurolysis: in addition to needle guidance, clinicians must reliably identify nerves and understand target relationships, which may require additional training and governance*
3. Evaluation of muscle structure, thickness, and composition to aid clinical assessment and decision making. *The third scope extends ultrasound beyond procedures into tissue characterisation and decision support—such as echogenicity grading (e.g., mHeckmatt), architectural assessment, and (where available) elastography—to provide additional information about MTU structure and composition that may contribute to interpretation of the relative neural and structural components of impairment and multidisciplinary treatment planning*

* An addition since the original publication of the statements.

**Table 3:** Ultrasound evaluation of muscle structure, thickness, and composition to aid clinical assessment and decision making in spasticity and contracture management.

Ultrasound Method	What Is Measured	Typical Findings in Spastic Muscle	Potential Clinical Relevance	Advantages	Limitations
Modified Heckmatt Scale (mHS) [30,49,50,51]	Semi-quantitative visual grading of muscle echogenicity (Grade 1–4) based on muscle brightness and visibility of deeper structures. After Rasch evaluation (original version) a restructured scale has been recommended rescoring to a 3-point scale, with response categories then consistently completed [49].	Higher grades indicate increased muscle echogenicity, which has been associated with fatty infiltration, fibrosis and other chronic structural muscle changes. Increased echogenicity is common after upper motor neuron lesions and chronic spasticity.	May provide additional information regarding muscle quality and the extent of structural change that can be considered alongside clinical assessment when establishing treatment goals and planning interventions. Its role in predicting responsiveness to BoNT or informing treatment selection has not yet been established prospectively. No definitive trials of decision making have been delivered.	Rapid, inexpensive, clinically feasible, good reliability and validity.	Subjective; dependent on operator experience and ultrasound settings. Validated operational thresholds and a decision-making framework to guide implementation are needed.
Architectural Assessment (B-mode Ultrasound) [35,52,53]	Muscle thickness, fascicle length, pennation angle, cross-sectional architecture, and muscle volume estimates.	Reduced muscle thickness, shorter fascicle length, altered pennation angle, and muscle atrophy may occur following stroke, cerebral palsy, spinal cord injury, and other spasticity conditions.	Provides information about muscle morphology and structural adaptation that may contribute to interpretation of the relative neural and structural components of impairment when considered alongside clinical assessment. Potential applications include treatment planning, longitudinal monitoring and evaluation of structural change. No definitive trials of decision making have been delivered.	Direct visualization of muscle morphology; widely available.	Measurement techniques require standardization; some parameters have moderate inter-rater reliability.
Elastography (Shear Wave or Strain Elastography) [31,32,54,55,56]	Quantitative assessment of tissue stiffness through measurement of shear-wave velocity or elasticity modulus.	Spastic muscles generally demonstrate increased stiffness compared with contralateral or healthy muscles. Significant differences have been demonstrated in biceps brachii and gastrocnemius muscles.	Provides quantitative information about tissue mechanical properties and may support monitoring of muscle changes over time or following intervention. Its potential contribution to interpreting neural and non-neural components of increased resistance to movement requires further clinical validation. No definitive trials of decision making have been delivered.	Objective, quantitative, sensitive to biomechanical changes.	Lack of standardized protocols; measurements affected by muscle position, loading, equipment, and operator technique.
Quantitative Grayscale Analysis (Echogenicity Analysis) [30,35,57,58]	Computer-assisted analysis of pixel intensity within a selected region of interest, usually reported as mean grayscale value.	Increased grayscale values indicate greater echogenicity associated with fibrosis, fatty infiltration, and loss of muscle quality. Quantitative grayscale correlates strongly with Modified Heckmatt scores.	Provides an objective measure of muscle echogenicity that may complement qualitative assessment and support longitudinal evaluation of muscle quality. Its value in predicting treatment response or informing selection for focal interventions remains to be established. No definitive trials of decision making have been delivered.	More objective than visual grading	Dependent on operator experience, ultrasound settings and appropriate equipment.

## Data Availability

No new data were created or analyzed in this study. Data sharing is not applicable to this article.

## Abbreviations

The following abbreviations are used in this manuscript:

AI	Artificial Intelligence
BoNT	Botulinum Toxin
CAST	Care of the Arm after STroke
mHeckmatt	Modified Heckmatt Scale
MTU	Muscle-Tendon Unit
PoCUS	Point-of-Care Ultrasound
NHS	National Health Service

## References

[1] Bernhardt J., Hayward K.S., Kwakkel G., Ward N.S., Wolf S.L., Borschmann K., Krakauer J.W., Boyd L.A., Carmichael S.T., Corbett D. (2017). Agreed definitions and a shared vision for new standards in stroke recovery research. Int. J. Stroke.

[2] Hunnicutt J.L., Gregory C.M. (2017). Skeletal muscle changes following stroke: A systematic review and comparison to healthy individuals. Top. Stroke Rehabil..

[3] Lieber R.L. (2010). Skeletal Muscle Structure, Function, and Plasticity: The Physiological Basis of Rehabilitation.

[4] Gillies A.R., Lieber R.L. (2011). Structure and function of the skeletal muscle extracellular matrix. Muscle Nerve.

[5] Reimers K., Reimers C.D., Wagner S., Paetzke I., Pongratz D.E. (1993). Skeletal muscle sonography: A correlative study of echogenicity and morphology. J. Ultrasound Med..

[6] Gao F., Grant T.H., Roth E.J., Zhang L.-Q. (2009). Changes in passive mechanical properties of the gastrocnemius muscle at the muscle fascicle and joint levels in stroke survivors. Arch. Phys. Med. Rehabil..

[7] Gao F., Zhang L.Q. (2008). Altered contractile properties of the gastrocnemius muscle poststroke. J. Appl. Physiol..

[8] Ada L., Dorsch S., Canning C.G. (2006). Strengthening interventions increase strength and improve activity after stroke: A systematic review. Aust. J. Physiother..

[9] Langhorne P., Bernhardt J., Kwakkel G. (2011). Stroke rehabilitation. Lancet.

[10] Gracies J.M. (2005). Pathophysiology of spastic paresis. I: Paresis and soft tissue changes. Muscle Nerve.

[11] Farmer S.E., James M. (2001). Contractures in orthopaedic and neurological conditions: A review of causes and treatment. Disabil. Rehabil..

[12] Pandyan A.D., Gregoric M., Barnes M.P., Wood D., Van Wijck F., Burridge J., Hermens H., Johnson G.R. (2005). Spasticity: Clinical perceptions, neurological realities and meaningful measurement. Disabil. Rehabil..

[13] Asci F., Falletti M., Zampogna A., Patera M., Hallett M., Rothwell J., Suppa A. (2023). Rigidity in Parkinson’s disease: Evidence from biomechanical and neurophysiological measures. Brain.

[14] Ditroilo M., Watsford M., Murphy A., De Vito G. (2011). Assessing musculo-articular stiffness using free oscillations: Theory, measurement and analysis. Sports Med..

[15] Kwah L.K., Harvey L.A., Diong J., Herbert R.D. (2012). Half of the adults who present to hospital with stroke develop at least one contracture within six months: An observational study. J. Physiother..

[16] Sackley C., Brittle N., Patel S., Ellins J., Scott M., Wright C., Dewey M.E. (2008). The prevalence of joint contractures, pressure sores, painful shoulder, other pain, falls, and depression in the year after a severely disabling stroke. Stroke.

[17] Malhotra S., Pandyan A.D., Rosewilliam S., Roffe C., Hermens H. (2011). Spasticity and contractures at the wrist after stroke: Time course of development and their association with functional recovery of the upper limb. Clin. Rehabil..

[18] Tariq H., Collins K., Tait D., Dunn J., Altaf S., Porter S. (2023). Factors associated with joint contractures in adults: A systematic review with narrative synthesis. Disabil. Rehabil..

[19] Allison R., Kilbride C., Chynoweth J., Creanor S., Frampton I., Marsden J. (2018). What is the longitudinal profile of impairments and can we predict difficulty caring for the profoundly affected arm in the first year poststroke?. Arch. Phys. Med. Rehabil..

[20] O’Dwyer N.J., Ada L., Neilson P.D. (1996). Spasticity and muscle contracture following stroke. Brain.

[21] Bavikatte G., Subramanian G., Ashford S., Allison R., Hicklin D. (2021). Early identification, intervention and management of post-stroke spasticity: Expert consensus recommendations. J. Cent. Nerv. Syst. Dis..

[22] Ashford S., Singer B., Rose H., Turner-Stokes L. (2022). The impact of spasticity and contractures on dependency and outcomes from rehabilitation. J. Int. Soc. Phys. Rehabil. Med..

[23] Katalinic O.M., Harvey L.A., Herbert R.D. (2011). Effectiveness of stretch for the treatment and prevention of contractures in people with neurological conditions: A systematic review. Phys. Ther..

[24] College of Occupational Therapists, Association of Chartered Physiotherapists in Neurology (2015). Splinting for the Prevention and Correction of Contractures in Adults with Neurological Dysfunction: Practice Guideline for Occupational Therapists and Physiotherapists.

[25] Ashford S.A., Morris G., Smith M.J. (2023). Ultrasound image guided injection of botulinum toxin for the management of spasticity: A Delphi study to develop recommendations for a scope of practice, competency, and governance framework. Arch. Rehabil. Res. Clin. Transl..

[26] Pillen S., van Alfen N. (2011). Skeletal muscle ultrasound. Neurol. Res..

[27] Shiina T., Nightingale K.R., Palmeri M.L., Hall T.J., Bamber J.C., Barr R.G., Castera L., Choi B.I., Chou Y.-H., Cosgrove D. (2015). WFUMB guidelines and recommendations for clinical use of ultrasound elastography: Part 1: Basic principles and terminology. Ultrasound Med. Biol..

[28] Pillen S., Arts I.M.P., Zwarts M.J. (2008). Muscle ultrasound in neuromuscular disorders. Muscle Nerve.

[29] Heckmatt J.Z., Leeman S., Dubowitz V. (1982). Ultrasound imaging in the diagnosis of muscle disease. J. Pediatr..

[30] Moreta M.C., Fleet A., Reebye R., McKernan G., Berger M., Farag J., Munin M.C. (2020). Reliability and validity of the modified Heckmatt scale in evaluating muscle changes with ultrasound in spasticity. Arch. Rehabil. Res. Clin. Transl..

[31] Wu C.-H., Ho Y.-C., Hsiao M.-Y., Chen W.-S., Wang T.-G. (2017). Evaluation of Post-Stroke Spastic Muscle Stiffness Using Shear Wave Ultrasound Elastography. Ultrasound Med. Biol..

[32] Zúñiga L.D.O., López C.A.G., González E.R. (2021). Ultrasound elastography in the assessment of the stiffness of spastic muscles: A systematic review. Ultrasound Med. Biol..

[33] Lehoux M.-C., Sobczak S., Cloutier F., Charest S., Bertrand-Grenier A. (2020). Shear wave elastography potential to characterize spastic muscles in stroke survivors: Literature review. Clin. Biomech..

[34] Campanella W., Corazza A., Puce L., Privitera L., Pedrini R., Mori L., Boccuni L., Turtulici G., Trompetto C., Marinelli L. (2022). Shear wave elastography combined with electromyography to assess the effect of botulinum toxin on spastic dystonia following stroke: A pilot study. Front. Neurol..

[35] Boissonnault È., Jeon A., Munin M.C., Filippetti M., Picelli A., Haldane C., Reebye R. (2024). Assessing muscle architecture with ultrasound: Implications for spasticity. Eur. J. Transl. Myol..

[36] Ko Y.D., Yun S.I., Ryoo D., Chung M.E., Park J. (2020). Accuracy of ultrasound-guided and non-guided botulinum toxin injection into neck muscles involved in cervical dystonia: A cadaveric study. Ann. Rehabil. Med..

[37] Henzel M.K., Munin M.C., Niyonkuru C., Skidmore E.R., Weber D.J., Zafonte R.D. (2010). Comparison of surface and ultrasound localization to identify forearm flexor muscles for botulinum toxin injections. PM&R.

[38] Heslot C., Khan O., Schnitzler A., Haldane C., David R., Reebye R. (2024). Enhancing botulinum toxin injection precision: The efficacy of a single cadaveric ultrasound training intervention for improved anatomical localization. Toxins.

[39] Matsumoto M.E., Berry J., Yung H., Matsumoto M., Munin M.C. (2018). Comparing electrical stimulation with and without ultrasound guidance for phenol neurolysis to the musculocutaneous nerve. PM&R.

[40] Korupolu R., Malik A., Pemberton E., Stampas A., Li S. (2022). Phenol neurolysis in people with spinal cord injury: A descriptive study. Spinal Cord. Ser. Cases.

[41] Guynn C.C. (2025). Percutaneous cryoneurolysis as a dynamic treatment for spasticity: A case series. Arch. Rehabil. Res. Clin. Transl..

[42] Pick A., Dye R., Fleming M.K. (2025). Cryoneurolysis: A novel treatment for management of spasticity. Presentation of a case series. Adv. Rehabil. Sci. Pract..

[43] Hashemi M., MacRae F., Boissonnault È., Vincent D., Song J., Shi S., Winston P. (2025). Measuring the efficacy of percutaneous cryoneurolysis in participants with refractory or plateaued shoulder, elbow, wrist, or finger spasticity. Am. J. Phys. Med. Rehabil..

[44] Santamato A., Micello M.F., Panza F., Fortunato F., Baricich A., Cisari C., Pilotto A., Logroscino G., Fiore P., Ranieri M. (2014). Can botulinum toxin type A injection technique influence the clinical outcome of patients with post-stroke upper limb spasticity? A randomized controlled trial comparing manual needle placement and ultrasound-guided injection techniques. J. Neurol. Sci..

[45] Hauret I., Dobija L., Givron P., Goldstein A., Pereira B., Coudeyre E. (2023). Effectiveness of ultrasound-guided vs electrical-stimulation-guided botulinum toxin injections in triceps surae spasticity after stroke: A randomized controlled study. J. Rehabil. Med..

[46] Esquenazi A., Zorowitz R.D., Ashford S., Beneteau M., Maisonobe P., Hannes C., Jacinto J. (2025). Longitudinal goal attainment with repeat injections of abobotulinumtoxinA in adults with lower limb spasticity: Results from a prospective observational study. Arch. Phys. Med. Rehabil..

[47] Fheodoroff K., Jacinto J., Ashford S., Danchenko N., Grandoulier A.-S., Pietri G., Bourhis Y., Spurden D., Turner-Stokes L. (2024). Predictors of real-world response in adults treated with botulinumtoxin-A for upper limb spasticity. J. Int. Soc. Phys. Rehabil. Med..

[48] Ashford S.A., Morris G., Alfonso J., Steed A., Smith M.J. (2026). Ultrasound image guided injection of botulinum toxin for the management of spasticity: An implementation case study for practice recommendations and a governance framework. Disabil. Rehabil..

[49] Wijntjes J., Saris C.G.J., Doorduin J., van Alfen N., van Engelen B.G.M., Mul K. (2024). Improving Heckmatt muscle ultrasound grading scale through Rasch analysis. Neuromuscul. Disord..

[50] Lagarde M.L.J., van den Engel-Hoek L., Geurts A.C.H., van Alfen N. (2023). Validity and reliability of visual assessment of orofacial muscle ultrasound images using a modified Heckmatt scale. Muscle Nerve.

[51] Asadi B., Pujol-Fuentes C., Carcasona-Otal A., Calvo S., Herrero P., Lapuente-Hernández D. (2024). Characterizing muscle tissue quality post-stroke: Echovariation as a clinical indicator. J. Clin. Med..

[52] Bonomi S., Palestini R., Cuccagna C., Ronconi G., Scaglione A., Oliva F., Fusco A. (2026). Ultrasound assessment of lower-limb muscle architecture in severe acquired brain injury: Prospective study. Muscles Ligaments Tendons J..

[53] Kong K.H., Tham S.-L., Tay M.R.J., Lui W.L., Rajeswaran D.K., Kim J. (2022). Ultrasound assessment of changes in muscle architecture of the brachialis muscle after stroke—A prospective study. Arch. Rehabil. Res. Clin. Transl..

[54] Illomei G. (2016). Muscle elastography in multiple sclerosis spasticity. Neurodegener. Dis. Manag..

[55] Kim J.H., Oh S.J., Kim S.Y., Kim T.U., Kim Y. (2026). Clinical validity of shear wave elastography for post-stroke spasticity: A systematic review and meta-analysis. J. Clin. Med..

[56] Roots J., Trajano G.S., Fontanarosa D. (2022). Ultrasound elastography in the assessment of post-stroke muscle stiffness: A systematic review. Insights Imaging.

[57] Tran A., Gao J. (2021). Quantitative ultrasound to assess skeletal muscles in post stroke spasticity. J. Cent. Nerv. Syst. Dis..

[58] Gao J., Memmott B., Poulson J. (2019). Quantitative ultrasound imaging to assess skeletal muscles in adults with multiple sclerosis: A feasibility study. Ultrasound Med. Biol..

[59] MacRae F., Boissonnault È., Lo A., Finlayson H., Winston P., Khan O., Dow H., Kassam F., Reebye R. (2024). Canadian physicians’ use of ultrasound in spasticity treatment: A national cross-sectional survey. Arch. Rehabil. Res. Clin. Transl..

[60] Cormican A., Hirani S.P., McKeown E. (2023). Healthcare professionals’ perceived barriers and facilitators of implementing clinical practice guidelines for stroke rehabilitation: A systematic review. Clin. Rehabil..

[61] Moore C.L., Copel J.A. (2011). Point-of-care ultrasonography. N. Engl. J. Med..

[62] Bahner D.P., Jasne A., Boore S., Mueller A., Cortez E. (2012). The ultrasound challenge: A novel approach to medical student ultrasound education. J. Ultrasound Med..

[63] Amini R., Stolz L.A., Breshears E., Patanwala A.E., Stea N., Hawbaker N., Thompson M., Sanders A.B., Adhikari S. (2017). Assessment of ultrasound-guided procedures in preclinical years. Intern. Emerg. Med..

[64] Tuvali O., Sadeh R., Kobal S., Yarza S., Golan Y., Fuchs L. (2020). The long-term effect of short point of care ultrasound course on physicians’ daily practice. PLoS ONE.

[65] Hoppmann R.A., Rao V.V., Poston M.B., Howe D.B., Hunt P.S., Fowler S.D., Paulman L.E., Wells J.R., Richeson N.A., Catalana P.V. (2011). An integrated ultrasound curriculum (iUSC) for medical students: 4-year experience. Crit. Ultrasound J..

[66] Damschroder L.J., Aron D.C., Keith R.E., Kirsh S.R., Alexander J.A., Lowery J.C. (2009). Fostering implementation of health services research findings into practice: A consolidated framework for advancing implementation science. Implement. Sci..

[67] Adhikari S., Amini R., Stolz L.A., Blaivas M. (2014). Impact of point-of-care ultrasound on quality of care in clinical practice. Rep. Med. Imaging.

[68] van Sloun R.J.G., Cohen R., Eldar Y.C. (2020). Deep learning in ultrasound imaging. Proc. IEEE.

